# Antiaging Effect of* Inula britannica* on Aging Mouse Model Induced by D-Galactose

**DOI:** 10.1155/2016/6049083

**Published:** 2016-03-15

**Authors:** Hui Chen, Yuanyuan Long, Lei Guo

**Affiliations:** Department of Burn and Plastic Surgery, The First Affiliated Hospital of Chongqing Medical University, Chongqing 400016, China

## Abstract

The antiaging effect of* Inula britannica* flower total flavonoids (IBFTF) on aging mice induced by D-galactose and its mechanism was examined in this study. From the results, the biochemical indexes and histological analysis of skin tissues showed that IBFTF could effectively improve the antioxidant enzyme activity of the aging mice, enhance the activities of superoxide dismutase (SOD), catalase (CAT), and glutathione peroxidase (GSH-Px) of skin tissue, and decrease the malondialdehyde (MDA) content. Besides, IBFTF could maintain the skin collagen, hydroxyproline (Hyp), dermal thickness, and moisture content. Meanwhile, IBFTF could significantly reduce the number of cells arrested in G0/G1 phase, and from the point of view of protein and mRNA expression level in skin tissue, IBFTF could significantly increase the expression of Sirt1 and CyclinD1 but decrease the expression of p16 and p21, and its effect was not less than that of the well-known vitamin E (VE). Overall, these results seem to be implying that IBFTF is a potential natural anti-skin aging agent with great antioxidant ability.

## 1. Introduction

From the biological viewpoint, aging is an inevitable spontaneous process and complex natural phenomenon [1]. In the aging process, the skin changes were most easily observed, which mainly performs as follows: gloomy skin, relaxation, moisture reduction, thinning, and so on. There are many factors that cause aging, including telomere shortening, oncogene activation, and DNA damage caused by reactive oxygen species (ROS), among which the most critical factor is the imbalance of oxygen free radical metabolism [2]. Free radical theory holds that because of the imbalance of free radical metabolism, the structure and function of tissues and organs are in disorder, which causes the body aging [3]. There are two signaling pathways associated with aging, p53-p21-Rb pathway and p16-pRb pathway [4].

An aging mouse model induced by D-galactose has been recognized by domestic and international researchers and widely used in the field of antiaging medicine research [5]. The aging degree of the model was close to 16–24 months of mouse, and all these changes were consistent with natural aging [6].

Because of its low toxicity, high efficiency, and low cost, more and more people prefer to choose pure natural plant agents to delay aging.* Inula britannica* flower has many biological activities such as antioxidant [7, 8], antidiabetes [9], immune regulation [10], antihepatitis [7], and antitumor [11], and its antioxidant activity is related with flavonoids [12]. Wang et al. [13] established a neuronal cell oxidative stress model and confirmed that icariin (components of traditional Chinese medicine) could decrease oxidative stress produced by ROS through upregulating the expression of antioxidant enzyme dependent on SIRT1. However, it is not clear whether the IBFTF is related to p21, p16, and Sirt1 or not.

To test our hypothesis, we extracted IBFTF, used in skin aging model induced by D-gal; then we observed the skin aging changes and explored the possible mechanism, which will lay the foundations for the development and application of IBFTF in delaying skin aging.

## 2. Materials and Methods

### 2.1. Plant Material

The plant material was dried flower of* Inula britannica* obtained from Chinese herbal medicine market in Chongqing (Changchun, Jiangsu, China) and authenticated by Professor Weiguo Cao (College of Traditional Chinese Medicine, Chongqing Medical University, China).

### 2.2. Reagents

All the chemicals and reagents were of analytical grade. D-galactose was purchased from Sigma-Aldrich (St Louis, USA). Commercial kits used for determination of SOD, MDA, GSH-Px, CAT, Hyp, and protein concentration were purchased from Nanjing Jiancheng Bioengineering Institute (Nanjing, China). Sirt1 and p16 antibodies were purchased from proteintech (Wuhan, China). P21 and CyclinD1 antibodies were purchased from Wanleibio (Shenyang, China). Trizol reagent, reverse transcription kit, and fluorescence quantitative reaction kit were purchased from TaKaRa (Dalian, China).

### 2.3. Animal Model and Treatment [6, 7, 14–17]

Kunming mice (SPF grade, male, 20 ± 2 g) was purchased from the Experimental Animal Center of Chongqing Medical University and the study was approved by the Ethics Committee of Chongqing Medical University. The mice were randomly divided into 6 groups (10 mice in each group). Except the control group subcutaneously injected with 0.3 mL saline, the other groups were injected with the same volume of D-galactose (500 mg/kg), once daily for 8 week. Followed by treatment with 0.5 mL 100, 200, or 400 mg/kg IBFTF or a positive control (100 mg/kg vitamin E) via intragastric administration once daily for 6 weeks from the third week. Control group and model group were orally given equal volume of saline once daily for 6 weeks. After 8 weeks, the mice were sacrificed following a 24-hour fasting period. The mice were sacrificed after 4% chloral hydrate anesthesia and high concentration of carbon dioxide, and then blood was collected and centrifuged under 4°C, 2000 r/min for 15 min. Blood serum was obtained and preserved at −20°C for standby. All the skin samples were taken from the injection site of the mice at the midline of the dorsum. Skin tissue was immediately collected and divided into two parts, one part was stored at −80°C, and the other part was fixed in 4% polyformaldehyde until further use.

### 2.4. Preparation of IBFTF [7]

The* Inula britannica* flower was boiled in water at 100°C for 1 h, and the extract was obtained by filtration. The total flavonoid extracts were purified using AB-8 resin under the following conditions: the extracted sample concentration was 20 mg/mL and washing was performed with 70% ethanol at a flow velocity of 2 mL/min.

### 2.5. Determination of IBFTF Content [7]

The extracts (1 mL) were mixed with 1 mL 5% NaNO_2_. After standing for 6 min, 1 mL 10% AlNO_3_ and 10 mL 4% NaOH were added to the mixture. The mixture was adjusted to 25 mL by adding 50% ethanol and allowed to rest for 15 min. Absorbance was measured at 510 nm. Rutin was used as a reference standard and the total flavonoids content was expressed relative to rutin. The result revealed that IBFTF content of* Inula britannica* flower was 82.6%.

### 2.6. Biochemical Analysis

The level of SOD, GSH-Px, CAT, and MDA was determined by kits. All procedures were performed according to the manufacture's instructions.

### 2.7. Measurement of Moisture Content

1 cm^2^ skin tissue was harvested and measured for the precise wet weigh, followed by baking at 80°C for 12 hours before measuring its dry weight. Skin water content was calculated by the formula of the percentage of water content = [(wet weight − dry weight)/wet weight] × 100%.

### 2.8. Measurement of Hydroxyproline Content

Hydroxyproline content in skin tissue was measured according to the manufacture's protocol of commercial assay kits.

### 2.9. Histological Examination

Skin tissue from mice of all six groups was fixed in 4% paraformaldehyde, dehydrated, and paraffin-embedded for haematoxylin and eosin (H&E) staining. The dermal thickness of the skin samples was measured with Image Pro Plus analysis software. To determine the amount of total collagen, samples obtained from all six groups were stained with Masson's trichrome. Total collagen content was calculated as a percentage of the aniline blue staining using Image Pro Plus analysis software.

### 2.10. Flow Cytometry

Hepatocyte suspension cells were made by liver tissue, fixed with precooled 70% ethanol at 4°C overnight, and then stained with propidium iodide containing RNase A at 37°C for 30 min in the dark. The cell cycle distribution was determined using a flow cytometer and the data were analyzed with Multicycle software.

### 2.11. RT-PCR Analysis

Total RNAs from skin tissues were isolated using Trizol reagent followed by TaKaRa Biotechnology (Dalian, China). cDNA was synthesized as recommended by TaKaRa Biotechnology (Dalian, China). The design and synthesis of specific primers of Sirt1, p16, p21, and CyclinD1 were completed by TaKaRa (Dalian, China). qRT-PCR was performed with the CFX96*™* real-time system (Bio-Rad, USA) using SYBR Premix Ex Taq*™*II (TaKaRa, Dalian, China). *β*-actin was internal reference. The sequences of the amplification primers were listed in Table 1. PCR conditions were as follows: 35 cycles of denaturation at 94°C for 30 sec, annealing at 57°C for 30 sec, and elongation at 72°C for 60 sec.

### 2.12. Western Blot Analysis

The skin tissue was cut into pieces and then ground in liquid nitrogen. Total protein from skin tissue was extracted with RIPA Lysis Buffer as described. The protein concentration was determined using the BCA Protein Assay Kit. *β*-actin served as a loading control. The protein bands were visualized using an Fusion software.

### 2.13. Statistical Analysis

Data were expressed as means ± SD. Statistical analysis was performed by one-way analysis of variance (ANOVA) using SPSS19.0 software. A value of *P* < 0.05 was considered to be statistically significant.

## 3. Results

### 3.1. Effect of IBFTF on Antioxidant Enzyme Activity in Aging Mice

In the model group, the activity of SOD in skin homogenate was lower than that in control group (*P* < 0.01), and the aging model was proved to build as expected successfully. The activity of SOD in the Vit E group and IBFTF groups was significantly higher than that in the model group (*P* < 0.01, resp.) but lower than that in the control group (*P* < 0.01); the SOD activity in the skin homogenate of low and medium dose of IBFTF group was lower than that in Vit E group (*P* < 0.05), while the difference between high dose IBFTF group and Vit E group was not statistically significant (Table 2).

In the model group, the content of MDA in skin homogenate was higher than that in control group (*P* < 0.01). The content of MDA in the Vit E group and IBFTF groups was significantly lower than that in the model group (*P* < 0.01) but higher than that in the control group (*P* < 0.01 or *P* < 0.05); the MDA content in the skin homogenate of low and medium dose of IBFTF group was higher than that in Vit E group (*P* < 0.05), while that in the high dose IBFTF group was lower than that in the Vit E group; the difference was not statistically significant (Table 2).

In the model group, the activity of CAT in skin homogenate was higher than that in control group (*P* < 0.01). The activity of CAT in the Vit E group and IBFTF groups was significantly lower than that in the model group (*P* < 0.01, resp.) but higher than that in the control group (*P* < 0.01, resp.). The CAT activity in the skin homogenate of low and medium dose of IBFTF group was lower than that in Vit E group (*P* < 0.05), while that in the high dose IBFTF group was higher than that in the Vit E group; the difference was not statistically significant (Table 2).

In the model group, the activity of GSH-Px in skin homogenate was higher than that in control group (*P* < 0.01). The activity of GSH-Px in the Vit E group and IBFTF groups was significantly lower than that in the model group (*P* < 0.01, resp.) but higher than that in the control group (*P* < 0.01, resp.); the GSH-Px activity in the skin homogenate of low and medium dose of IBFTF group was lower than that in Vit E group (*P* < 0.05), while that in the high dose IBFTF group was higher than that in the Vit E group; the difference was not statistically significant (Table 2).

### 3.2. Effect of IBFTF on Physical Properties of Skin Aging Induced D-Gal

**Figure 1:**
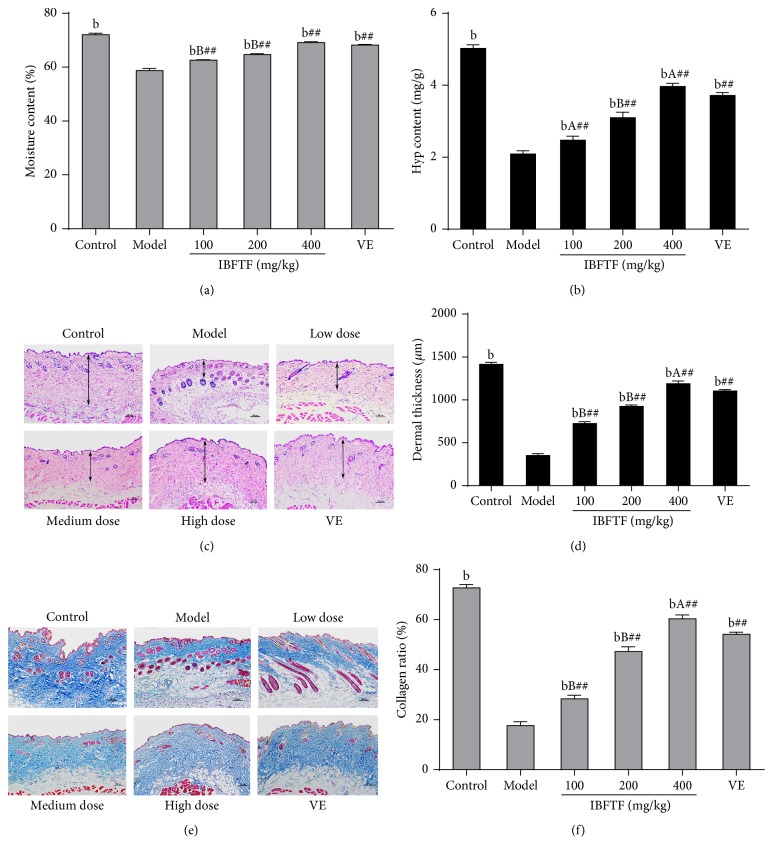
Physical properties of D-gal induced a skin aging after treatment. (a) The effects of EAE on the changes of moisture content. (b) The effects of EAE on the changes of Hyp content. (c) The IBFTF treatment increased dermal thickness (the double head arrows). (d) The thickness of the dermal portion of skin. (e) The IBFTF treatment increased collagen ratio of mice skin. (f) Collagen ratio (collagen fibers stained blue) was measured with an Image Pro Plus program. *n* = 4. Magnification: ×200, bar for 100 mm. Each value is the means ± SD (*n* = 8). ^b^ *P* < 0.01 compared with model group (all groups). ^A^ *P* < 0.05; ^B^ *P* < 0.01 compared with Vit E group (IBFTF groups). ^##^ *P* < 0.01 compared with control group (Vit E and IBFTF groups).

The skin moisture content in the model group was lower than that in the control group, and the difference was statistically significant (*P* < 0.01). The skin moisture content in the Vit E group and IBFTF groups was significantly higher than that in model group (*P* < 0.01, resp.) but lower than that in control group (*P* < 0.01, resp.). The skin moisture content in low dose and medium dose IBFTF group was significantly lower than that in Vit E group (*P* < 0.01), and the skin moisture content of high dose IBFTF group was higher than that in Vit E group, and the difference was not statistically significant (Figure 1(a)).

The skin Hyp content in the model group was lower than that in the control group, and the difference was statistically significant (*P* < 0.01). The skin Hyp content in the Vit E group and IBFTF groups was significantly higher than that in model group (*P* < 0.01, resp.) but lower than that in control group (*P* < 0.01, resp.). The skin Hyp content in low dose and medium dose IBFTF group was significantly lower than that in Vit E group (*P* < 0.05), while the skin Hyp content of high dose IBFTF group was higher than that in Vit E group (*P* < 0.05), and low, medium, and high dose IBFTF groups increased the Hyp content of the model group in a dose dependent manner (Figure 1(b)).

The dermal thickness in the model group was lower than that in the control group, and the difference was statistically significant (*P* < 0.01). The dermal thickness in the Vit E group and IBFTF groups was significantly higher than that in model group (*P* < 0.01, resp.) but lower than that in control group (*P* < 0.01, resp.). The dermal thickness in low dose and medium dose IBFTF group was significantly lower than that in Vit E group (*P* < 0.01), while the dermal thickness of high dose IBFTF group was higher than that in Vit E group (*P* < 0.05) (Figures 1(c) and 1(d)).

The skin collagen fiber content in the model group was lower than that in the control group (*P* < 0.01). The skin collagen fiber content in the Vit E group and IBFTF groups was significantly higher than that in model group (*P* < 0.01, resp.) but lower than that in control group (*P* < 0.01, resp.). The skin collagen fiber content in low dose and medium dose IBFTF group was significantly lower than that in Vit E group (*P* < 0.01), while the skin collagen fiber content of high dose IBFTF group was higher than that in Vit E group (*P* < 0.05) (Figures 1(e) and 1(f)).

### 3.3. Effect of IBFTF on Cell Cycle

**Figure 2:**
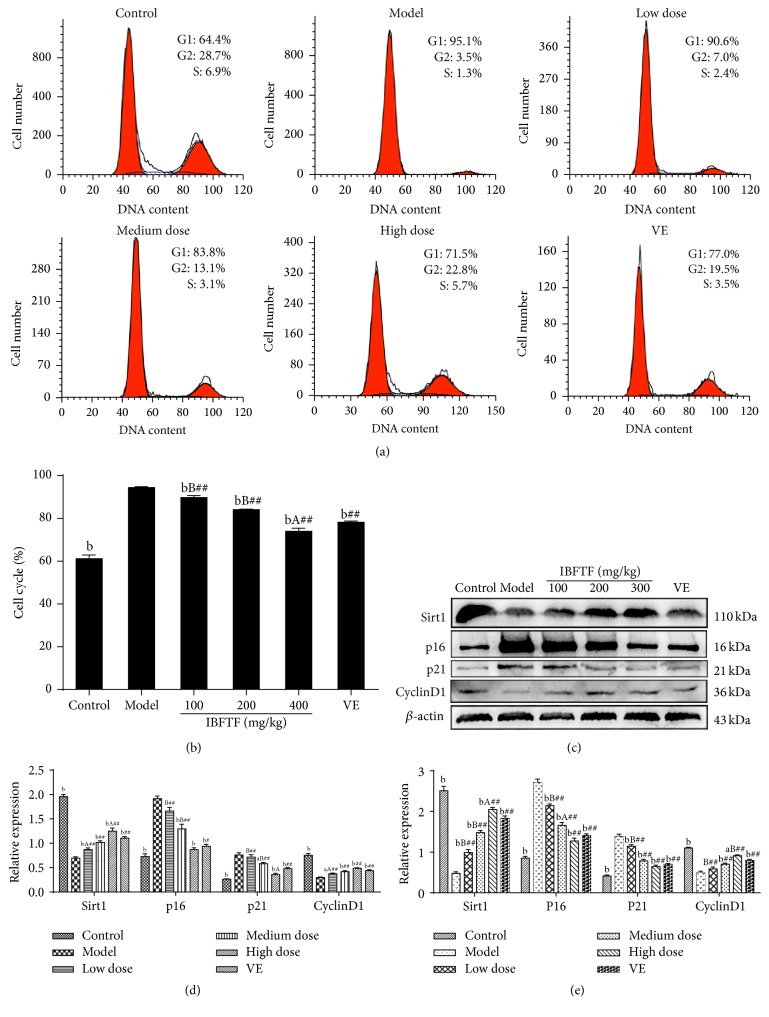
The IBFTF influences the expression of Sirt1 to indirect regulation of p16^INK4a^ and P53-P21^Cip1^ pathways. The cell cycle distribution of the cells was analyzed by flow cytometry. The representative graphs are shown in (a). The quantitative analysis is demonstrated as histograms in (b). The protein levels of regulators of cell cycle were detected by western blot in (c) and (d). (e) The mRNA levels of regulators of cell cycle were detected by RT-qPCR. The data are presented as means ± SD (*n* = 4) and all experiments were done in triplicate. ^a^ *P* < 0.05; ^b^ *P* < 0.01 compared with model group (all groups). ^A^ *P* < 0.05; ^B^ *P* < 0.01 compared with Vit E group (IBFTF groups). ^#^ *P* < 0.05; ^##^ *P* < 0.01 compared with control group (Vit E and IBFTF groups).

Cell cycle distribution was detected by flow cytometry, and cells were labeled with PI and annexin V. The number of cells arrested in G0/G1 phase in the model group was significantly lower than that in the control group, and the difference was statistically significant (*P* < 0.01). The number of cells arrested in G0/G1 phase in the Vit E group and IBFTF groups was significantly lower than that in the model group (*P* < 0.01, resp.) but significantly higher than that in the control group (*P* < 0.01). The number of cells arrested in G0/G1 phase of low and medium dose of IBFTF group was significantly higher than that in Vit E group (*P* < 0.01), while the number of cells in high dose group was lower than that in Vit E group (*P* < 0.05). Low, medium, and high dose IBFTF reduced the number of cells arrested in G0/G1 phase of the model group in a dose dependent manner (Figures 2(a) and 2(b)).

### 3.4. Effect of IBFTF on mRNA Expression of Sirt1, p16, p21, and CyclinD1 Target Genes

In the model group, the mRNA expression of Sirt1 in the skin was lower than that in the control group (*P* < 0.01), and mRNA expression of Sirt1 of the Vit E and IBFTF groups was significantly higher than that in the model group (*P* < 0.01, resp.) but lower than that in the control group (*P* < 0.01, resp.); mRNA expression of Sirt1 in skin tissues of low and medium dose IBFTF group was lower than that in Vit E group (*P* < 0.01, resp.); mRNA expression of Sirt1 in high dose IBFTF group was higher than that in Vit E group (*P* < 0.05). Low, medium, and high dose IBFTF increased the mRNA expression of Sirt1 in the skin tissue of the model group in a dose dependent manner (Figure 2(e)).

In the model group, mRNA expression of p16 in the skin was lower than that in the control group (*P* < 0.01); mRNA expression of p16 of the Vit E group and IBFTF groups was significantly lower than that in the model group (*P* < 0.01, resp.) but higher than that in the control group (*P* < 0.01, resp.); mRNA expression of p16 in skin tissues of low and medium dose IBFTF group was higher than that in Vit E group (*P* < 0.05); mRNA expression of p16 in high dose IBFTF group was lower than that in Vit E group; the difference was not statistically significant (Figure 2(e)).

In the model group, mRNA expression of p21 in the skin was lower than that in the control group (*P* < 0.01). mRNA expression of p21 of the Vit E and IBFTF groups was significantly lower than that in the model group (*P* < 0.01, resp.) but higher than that in the control group (*P* < 0.01, resp.); mRNA expression of p21 in skin tissues of low and medium dose IBFTF group was higher than that in Vit E group, and the difference between the low dose IBFTF group and Vit E group was statistically significant (*P* < 0.01). The mRNA expression of p21 in high dose IBFTF group was lower than that in Vit E group; the difference was not statistically significant (Figure 2(e)).

In the model group, the mRNA expression of CyclinD1 in the skin was lower than that in the control group (*P* < 0.01). The mRNA expression of CyclinD1 of the Vit E group and IBFTF groups was significantly higher than that in the model group (*P* < 0.01, resp.) but lower than that in the control group (*P* < 0.01, resp.). The mRNA expression of CyclinD1 in skin tissues of low and medium dose IBFTF group was lower than that in Vit E group, and the difference between the low dose IBFTF and Vit E group was statistically significant (*P* < 0.01). The mRNA expression of CyclinD1 in high dose IBFTF group was higher than that in Vit E group (Figure 2(e)).

### 3.5. Effect of IBFTF on Protein Expression of p16, Sirt1, CyclinD1, and p21

By detecting the expression of some key proteins (p16^INK4a^ and P21^Cip1^) in the senescence-associated signaling pathway, the protein expression of Sirt1 in skin tissue of the model group was lower than that in the control group (*P* < 0.01). The protein expression of Sirt1 in the skin tissues of Vit E group and IBFTF groups was significantly higher than that in the model group (*P* < 0.01, resp.) but significantly lower than that in the control group (*P* < 0.01, resp.). The protein expression of Sirt1 in skin tissues of low and medium dose IBFTF group was lower than that in Vit E group, and the difference between the low IBFTF and Vit E group was statistically significant (*P* < 0.05). The protein expression of Sirt1 in high dose IBFTF group was higher than that in Vit E group (*P* < 0.05) (Figures 2(c) and 2(d)).

The protein expression of p16 in skin tissue of the model group was higher than that of control group, and the difference was statistically significant (*P* < 0.01). The protein expression of p16 in the skin tissues of Vit E group and IBFTF groups was significantly lower than that in the model group (*P* < 0.01, resp.). The protein expression of p16 in skin tissues of low and medium dose IBFTF group was higher than that in control group (*P* < 0.01). The protein expression of p16 in high dose IBFTF group was higher than that in control group, but there was no significant difference. The protein expression of p16 in skin tissues of low and medium dose IBFTF group was higher than that in Vit E group (*P* < 0.01), while the protein expression of p16 in high dose IBFTF group was lower than that in Vit E group, and the difference was not statistically significant (Figures 2(c) and 2(d)).

The protein expression of p21 in skin tissue of the model group was higher than that of control group (*P* < 0.01). The protein expression of p21 in the skin tissues of Vit E group and IBFTF groups was significantly lower than that in the model group, and the difference among the medium dose, high dose IBFTF and Vit E group was statistically significant (*P* < 0.05, resp.). The protein expression of p21 in skin tissues of low dose, medium dose IBFTF and Vit E group was higher than that in control group (*P* < 0.01, resp.). The protein expression of p21 in high dose IBFTF group was higher than that in control group, but there was no significant difference. The protein expression of p21 in skin tissues of low and medium dose IBFTF group was higher than that in Vit E group (*P* < 0.01), while the protein expression of p21 in high dose IBFTF group was lower than that in Vit E group (*P* < 0.05) (Figures 2(c) and 2(d)).

The protein expression of CyclinD1 in skin tissue of the model group was lower than that of control group, and there was a significant difference (*P* < 0.01). The protein expression of CyclinD1 in the skin tissues of Vit E group and IBFTF groups was significantly higher than that in the model group (*P* < 0.05, resp.) but significantly lower than that in the control group (*P* < 0.01, resp.). The protein expression of CyclinD1 in skin tissues of low and medium dose IBFTF group was lower than that in Vit E group, and the difference between low dose IBFTF and Vit E group was statistically significant (*P* < 0.05). The protein expression of CyclinD1 in high dose IBFTF group was higher than that in Vit E group, but there was no significant difference (Figures 2(c) and 2(d)).

## 4. Discussion

With the increase of age, the activity of antioxidative enzymes decreased in vivo, such as SOD, CAT, and GSH-Px, and the ability of scavenging free radicals decreased, leading to free radical chain reaction and eventually causing lipid peroxidation end-product of malondialdehyde (MDA) increased [18]. Compared with the model group, the antioxidant activity of Vit E and IBFTF groups was significantly improved, and MDA was significantly decreased in a dose dependent manner, but there was no significant difference between Vit E and the high dose IBFTF group. It indicates that IBFTF may delay senescence by upregulating the expression of the antioxidant enzymes relying on Sirt1 in a dose dependent manner.

Skin can keep smooth and delicate mainly depending on the content of dermal collagen. The skin Hyp content rich or not can directly reflect the change of collagen fiber content, so as to reflect the degree of skin aging [19]. In addition, moisture content of the skin plays an important role in maintaining normal function of the skin [20]. In our experiment, the content of collagen fibers, Hyp, and moisture in the skin tissue of the model group were significantly lower than those in the control group and IBFTF groups. This indicates that IBFTF can delay skin aging by improving the content of collagen fibers, Hyp, and moisture content in the model group.

The mechanism of delaying skin aging by traditional Chinese medicine has focused on scavenging free radical and antioxidant activity. Studies have shown that P16 is the key link in the genetic control of cellular senescence program. The main function of P16 is to inhibit the phosphorylation of CyclinD-CDK4/6-RB specifically, so that the cell growth was arrested in the G1 phase, which leads to the phenomenon of aging [21]. P21 is a broad spectrum of cell cycle protein and an inhibitor depending on cell cycle protein kinase (CDK), which is involved in cell growth, differentiation, aging, and apoptosis by regulating cell cycle progression [22]. CyclinD1 is an important positive regulatory molecule in G1/S phase, which has an important effect on cell proliferation [23]. The upregulation of P21 and P16 levels can inhibit the activity of CyclinD1, so that the cell can not enter S phase, eventually leading to cell cycle arrest [24]. No matter what kind of factors causing aging, it will eventually be settled on the regulation of cell cycle. SIRT1 is highly conserved NAD^+^-dependent protein deacetylases, plays its function of gene regulation through interactions with the key protein of signaling pathways, and involves cell senescence and apoptosis and oxidative stress reaction process [4]. Yuan et al. [4] explored the effect and mechanism of Sirt1 in the mesenchymal stem cells by intervening the expression of Sirt1. The results showed that the upregulation of P16 was followed by the expression of cell senescence of MSCs in bone marrow induced by the downregulation of SIRT1, while the change of P21 was not obvious; the expression of cell senescence of fat-derived MSCs after downregulation of SIRT1 will be accompanied by the upregulation of P16 and P21. This result suggests that the aging mechanism of MSCs is not the same in different tissues. The experimental results showed that cells in the model group were arrested in G0/G1 phase obviously compared with control group, while the percentage of G0/G1 in IBFTF groups was significantly lower than that in the model group. From the results of western blot and qRT-PCR, compared with model group, we found that the expression of CyclinD1 and Sirt1 in the skin tissues of the IBFTF groups significantly increased, while the expression of p21 and p16 decreased. The above results suggested that IBFTF may delay the aging process of skin via affecting the expression level of Sirt1 to indirectly regulate p16^INK4a^ and P53-P21^Cip1^ pathways. All these data indicated that antiaging effect of IBFTF in aging mice may be dependent on the regulation of Sirt1.

## 5. Conclusions 

In summary, the results indicate that antiaging effect of IBFTF in aging mice depended on the regulation of Sirt1 gene in a dose dependent manner.

## Figures and Tables

**Table 1:** Primer pairs for target and housekeeping genes for quantitative RT-PCR assay.

Target gene accession	Forward	Sequence Reverse	Probe length
P16 NM_001040654.1	TGCTCAACTACGGT GCAGATTC	ATGTCTTGATGTCCC CGCTCT	190 bp
P21 NM_001111099.1	CCAATCCTGGTGAT GTCCGA	AGTCAAAGTTCCACC GTTCTCG	150 bp
CyclinD1 NM_007631.2	GTGAGGAGCAGAA GTGCGAAGA	GGCCGGATAGAGTTG TCAGTGTAG	199 bp
SirT1 NM_001159589	AGGGAACCTTTGCC TCATCTAC	GTTTGGCATATTCACC ACCTAGC	103 bp
*β*-actin NM_007393.4	AGATTACTGCTCTG GCTCCTAGC	ACTCATCGTACTCCTGCT TGCT	147 bp

**Table 2:** Antioxidants status in the skin tissue of mice in each group.

Group	SOD (U/mg prot)	MDA (nmol/mg prot)	CAT (U/mg prot)	GSH-Px (nmol/mg prot)
Control	114.86 ± 6.54^b^	5.11 ± 0.64^b^	18.27 ± 1.18^b^	477.24 ± 21.61^b^
Model	66.38 ± 6.53	10.12 ± 1.65	9.04 ± 1.52	355.72 ± 23.95
Low dose	75.24 ± 9.38^bA##^	9.08 ± 0.58^bA##^	11.28 ± 0.97^bB##^	373.10 ± 17.40^B##^
Medium dose	86.69 ± 7.02^bA##^	7.77 ± 0.80^bB##^	13.03 ± 0.80^bA##^	397.39 ± 15.74^bB##^
High dose	100.44 ± 10.42^b##^	6.08 ± 0.44^b#^	14.92 ± 0.79^b##^	435.38 ± 19.28^b##^
Vitamin E	96.15 ± 6.47^b##^	6.26 ± 0.63^b##^	14.37 ± 1.32^b##^	430.48 ± 5.64^b##^

Values are the mean ± SD.

^b^ *P* < 0.01 compared with model group (all groups).

^A^ *P* < 0.05; ^B^ *P* < 0.01 compared with Vit E group (IBFTF groups).

^#^ *P* < 0.05; ^##^ *P* < 0.01 compared with control group (Vit E and IBFTF groups).
